# Expression of Potential Targets for Cell-Based Therapies on Melanoma Cells

**DOI:** 10.3390/life11040269

**Published:** 2021-03-24

**Authors:** Sophia B. Strobel, Devayani Machiraju, Ingrid Hülsmeyer, Jürgen C. Becker, Annette Paschen, Dirk Jäger, Winfried S. Wels, Michael Bachmann, Jessica C. Hassel

**Affiliations:** 1 Department of Dermatology and National Center for Tumor Diseases, University Hospital Heidelberg, 69120 Heidelberg, Germany; sophia.strobel@med.uni-heidelberg.de (S.B.S.); devayani.machiraju@med.uni-heidelberg.de (D.M.); 2 German Cancer Research Center (DKFZ), 69120 Heidelberg, Germany; i.huelsmeyer@dkfz-heidelberg.de (I.H.); j.becker@dkfz-heidelberg.de (J.C.B.); annette.paschen@uk-essen.de (A.P.); wels@gsh.uni-frankfurt.de (W.S.W.); 3 Translational Skin Cancer Research, German Cancer Consortium (DKTK), 45141 Essen, Germany; 4 Department of Dermatology, University Hospital Essen, 45147 Essen, Germany; 5 National Center for Tumor Diseases (NCT) Heidelberg, Department of Medical Oncology, Heidelberg University Hospital, 69120 Heidelberg, Germany; dirk.jaeger@med.uni-heidelberg.de; 6 National Center for Tumor Diseases, German Cancer Research Center, Clinical Cooperation Unit Applied Tumor Immunity, 69120 Heidelberg, Germany; 7 Georg-Speyer-Haus, Institute for Tumor Biology and Experimental Therapy, 60596 Frankfurt am Main, Germany; 8 German Cancer Consortium (DKTK), Partner Site Frankfurt/Mainz, 60590 Frankfurt am Main, Germany; 9 Frankfurt Cancer Institute, Goethe University, 60590 Frankfurt am Main, Germany; 10 Helmholtz-Zentrum Dresden-Rossendorf (HZDR), Institute of Radiopharmaceutical Cancer Research, 01328 Dresden, Germany; m.bachmann@hzdr.de; 11 German Cancer Consortium (DKTK), Partner Site Dresden, and German Cancer Research Center (DKFZ), 69120 Heidelberg, Germany; 12 National Center for Tumor Diseases (NCT), University Hospital ‘Carl Gustav Carus’, TU Dresden, 01307 Dresden, Germany; 13 Tumor Immunology, University Cancer Center (UCC) ‘Carl Gustav Carus’, TU Dresden, 01307 Dresden, Germany

**Keywords:** melanoma, target, HER2, TRP2, ABCB5, gp100, p53, GD2

## Abstract

Tumor antigen-specific redirection of cytotoxic T cells (CTLs) or natural killer (NK) cells including chimeric antigen receptor (CAR-) and T cell receptor (TCR-) cell therapy is currently being evaluated in different tumor entities including melanoma. Expression of melanoma-specific antigen recognized by the respective CAR or TCR directly or presented by HLA molecules is an indispensable prerequisite for this innovative therapy. In this study, we investigated in 168 FFPE tumor specimens of patients with stage I-IV melanoma the protein expression of HER2, TRP2, ABCB5, gp100, p53, and GD2 by immunohistochemistry (IHC). These results were correlated with clinical parameters. Membrane expression of HER2 and GD2 was also investigated in ten melanoma cell lines by flow cytometry for which corresponding tumors were analyzed by IHC. Our results demonstrated that gp100 was the most frequently overexpressed protein (61%), followed by TRP2 (50%), GD2 (38%), p53 (37%), ABCB5 (17%), and HER2 (3%). TRP2 expression was higher in primary tumors compared to metastases (*p* = 0.005). Accordingly, TRP2 and ABCB5 expression was significantly associated with lower tumor thickness of the primary (*p* = 0.013 and *p* = 0.025). There was no association between protein expression levels and survival in advanced melanoma patients. Flow cytometric analysis revealed abundant surface expression of GD2 and HER2 in all melanoma cell lines. The discordant HER2 expression in situ and in vitro suggests a tissue culture associated induction. In summary, our data support the use of gp100 and GD2 as a potential target for developing engineered TCR- or CAR-cell therapies, respectively, against melanoma.

## 1. Introduction

Immunotherapeutic approaches gain continuously increasing importance against cancer. In melanoma, immunotherapies with immune checkpoint blockers made it possible to even cure patients [1,2]. However, two thirds of patients with metastatic disease will eventually progress. More specific immunotherapies against the melanoma might be more efficacious. Tumor antigen-specific redirection of cytotoxic T cells (CTLs) including chimeric antigen receptor (CAR-) and T cell receptor (TCR-) T cell therapy showed huge success in hematological malignancies, and its efficacy is currently being evaluated in different solid tumors including melanoma [3,4,5]. Melanoma is a very aggressive form of skin cancer and is known for its immunogenic features [6]. Identification and selection of an ideal melanoma-associated antigen that can be recognized by the CTLs or natural killer (NK) cells remain a challenging step for a successful engineered cell-based therapy in melanoma.

In this regard, CTLs that recognize tumor-associated antigens (TAAs) like glycoprotein 100 (gp100), tyrosinase-related protein 2 (TRP2) were isolated from melanoma patients previously [7,8,9,10,11]. gp100 is an intracellular highly melanoma-specific protein involved in melanin synthesis [12,13]. In vitro, T cells expressing, gp100-specific TCR and chondroitin sulfate proteoglycan 4 (CSPG4)-specific CAR showed potent anti-tumor cytotoxicity against melanoma cells [14]. Similarly, TRP2, an intracellular melanogenic enzyme is expressed by both melanocytes and melanomas [15,16]. TRP2 vaccination in mice suggested that tolerance to TRP2 can be inhibited, and TRP2-specific CTLs can mediate anti-tumor immunity against melanoma [17,18]. Furthermore, the transcription factor p53 is considered to be a potential anti-cancer target. p53 plays an important role in response to cellular stress and is crucial in protection against cancer development [19]. However, the p53 gene is the most commonly mutated gene found in human malignancies preventing them from apoptosis [20,21]. A study demonstrated that antigen-experienced T cells from peripheral blood lymphocytes represent a source of T cells with specificity to p53-mutated neoantigens [22,23].

In addition, GD2 ganglioside is a glycosphingolipid involved in signal transduction, cell–cell recognition, and tumor cell metastasis, and is known to be overexpressed in solid tumors [24,25,26]. GD2-specific CAR-T lymphocytes exhibit potent anti-melanoma activity in vitro and in vivo [27]. Moreover, ATP-binding cassette subfamily B member 5 (ABCB5) expression preferentially marks a subset of hyperpolarized, CD133+ stem cell phenotype-expressing melanoma cells, and serves as a drug transporter and chemoresistance mediator in melanoma [28]. Finally, overexpression of HER2 (ErbB2), a transmembrane tyrosine kinase receptor protein was associated with increased basal tyrosine kinase activity that chronically stimulates signal transduction pathways, leading to malignant transformation of cells. Recently, it was shown that CAR-T cells directed against HER2 can kill uveal and cutaneous melanoma cells in vitro and in vivo [29]. However, expression levels might be low.

In this study, we aimed to characterize the expression and distribution of candidate antigens in melanoma that might be potential targets for cell-based therapies including gp100, TRP2, p53, GD2, ABCB5, and HER2.

## 2. Materials and Methods

### 2.1. Patients and Samples

Fifty patients were planned for analyzing in each melanoma stage (stage I, II, III, and IV). Two hundred archived formalin-fixed, paraffin-embedded (FFPE) tumor samples from melanoma patients diagnosed with primary or metastatic melanoma between 2012 and 2019 at the University Hospital Heidelberg, Department of Dermatology were picked. Since there was not enough material to process further for IHC in 3 samples, 197 samples were stained. Another 29 patients were excluded due to lack of tumor content, and finally, in total 168 patients were analyzed and included in the study. Diagnosis and tumor content were reevaluated by a dermatopathologist (JCH). Clinical data of the patients were retrieved from medical records. From patients, where primary melanoma cell lines had been established and archived in the liquid biobank of the Section of Dermato-Oncology that originated from the same melanoma biopsy, the primary melanoma cell line was used for fluorescence activated cell sorting (FACS) analysis. The study was approved by the ethics committee of Heidelberg University (S-091/2011; S-454/2015; S-207/2015).

### 2.2. Immunohistochemical Staining

The immunohistochemical stainings (IHC) were performed and provided by the Tissue Bank of the National Center for Tumor Diseases (NCT) Heidelberg, Germany (#2778), in accordance with the regulations of the tissue bank and the approval of the ethics committee of Heidelberg University (S-091/2011). Serial tissue sections of 2 μm were prepared from FFPE melanoma tissue blocks. Automated immunohistochemical staining was performed by using a fully automated stainer (Ventana Benchmark ULTRA, Roche Diagnostics, Basel, Switzerland). Sections were incubated with rabbit-polyclonal anti-ABCB5 (1:50; #NBP1-77687, Novus Biologicals, Littleton, CO, USA); mouse monoclonal anti-GD2(1:25; clone 14.G2a; #554272; BD Biosciences, Franklin Lakes, NJ, USA); rabbit monoclonal anti-HER2 (1:100; clone SP3; #237R; Cell Marque, Rocklin, CA, USA); mouse monoclonal anti-HMB45 (1:75; clone HMB-45; #M063429-2; DAKO Agilent, Santa Clara, CA, USA); mouse monoclonal anti-p53(1:400; clone DO-7; #M700101-2, DAKO Agilent, Santa Clara, CA, USA); and mouse monoclonal anti-TRP2 (1:5000; clone C-9; #sc-74439; Santa Cruz Biotech, Dallas, TX, USA) antibodies at 37 °C for 30 min, and the IHC reaction was detected using OptiView DAB IHC Detection Kit (Ventana, Roche Diagnostics, Basel, Switzerland) and diaminobenzidine (DAB) was used as a chromogen. Sections were counterstained with hematoxylin II and Bluing Reagent (Ventana, Roche Diagnostics, Basel Switzerland) and mounted with Shandon Consul-Mount (Thermo Fisher Scientific, Waltham, MA, USA) in a fully automated coverslipper (Leica CV5030, Leica Biosystems, Wetzlar, Germany). For analyzing the immunohistochemical expression a semiquantitative approach was used to generate an H-Score for each marker based on the intensity of the staining (0–3; 0-negative, 1-weak, 2-moderate, and 3-intense) and the percentage of tumor cells positively stained (0–100%) in the tissue [30]. The H-Score (0–300) was calculated by multiplying the percentage of the stained tumor area by the staining intensity. Samples with an H-score above the median were defined as “high“. Whereas, the HER2 IHC expression was defined as positive or negative based on its presence in tumor cells. All stained slides were reviewed and scored in a blinded manner.

### 2.3. Tumor Cell Lines

Tumor cell suspensions were prepared from melanoma metastasis by enzymatic digestion in collagenase (Gibco, Life Technologies, Carlsbad, CA, USA) with constant stirring for 30 min at 37 °C, followed by centrifugation. Cells were cultured in complete growth media consisted of RPMI 1640 (Gibco, Life Technologies, Carlsbad, CA, USA) supplemented with 10% heat-inactivated FBS, 2mM HEPES, 1mM ABAM, and 1mM Gentamycin. Melanoma cell line SK-MEL-28 was cultured in DMEM containing 10% FBS, 1mM sodium pyruvate, 1mM Pen/Strep, and human breast cancer cell lines KPL-4 and MCF-7 (kindly provided by Ms. Simone Jünger, DKFZ).

### 2.4. Immunophenotyping via Flow Cytometry

Tumor cells were washed in PBS with 2% FCS (FACS buffer) followed by incubation with a human Fc receptor blocking reagent (KIOVIG, Baxter, Deerfield, MA, USA). Live/Dead staining (Thermo Fisher Scientific, Waltham, MA, USA) was used to be able to distinguish live and dead cells. Cells were then stained with the extracellular fluorescent-labeled antibodies (BioLegend, San Diego, CA, USA) according to the cell panel: mouse monoclonal HER2 AF647(clone 24D2; #324412), mouse monoclonal GD2 PE (clone 14G2a; #357304), and corresponding Isotypes. After staining for surface markers, cells were washed twice with FACS buffer and analyzed on BD FACS Canto II (BD Biosciences, Franklin Lakes, NJ, USA) with antibody stained beads used for compensation (CompBeads, BD Biosciences, Franklin Lakes, NJ, USA). Data were analyzed using FlowJo software version 10 (Tree Star, BD Biosciences, Franklin Lakes, NJ, USA). Melanoma cells (live and single cells) were divided into HER2+ and GD2+ populations.

### 2.5. Statistical Analysis

All statistical analyses were carried out using SPSS version 25 (IBM, Ehningen, Germany). The relation between clinical parameters and differential expression of proteins was evaluated by chi-square testing. Progression-free survival (PFS) was measured from the start of any systemic treatment within 3 months of the biopsy until disease progression (PD) or death from melanoma, whereas overall survival (OS) was measured from the start of treatment until death from any cause. Patients without an event were censored at last contact. The Kaplan–Meier method was used to describe differences in survival for the different expression groups; statistical analysis was carried out by use of a log-rank test. *p* < 0.05 was considered to indicate a statistically significant difference.

## 3. Results

### 3.1. Patient Characteristics

In total, 168 melanoma tissues of 98 primary tumors and 70 metastases (52 skin metastases, 11 lymph node metastases, 7 visceral metastases) were evaluable in this study (see Table 1). Median age of the patients was 67 years (range: 30–94 years) with 98 (58%) male patients. Median Breslow thickness of the primary melanoma was 1.5 mm (range 0.2–17 mm) with 29% presenting with an ulcerated phenotype. From the ones with known BRAF status, 28 out of 71 patients revealed a BRAFV600 mutation (39.4%), among them 25 patients had BRAF V600E mutation and the other 3 had BRAF V600K mutation. LDH and S100 were obtained at the time of the biopsy at normal levels in 83%, respectively in 78.6% of the patients.

### 3.2. Immunohistochemical Expression of Target Proteins in Primary and Advanced Melanoma

**Figure 1:**
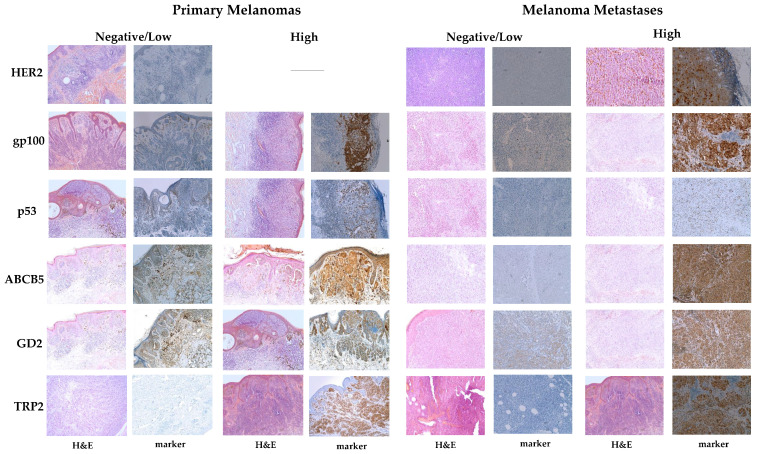
Representative immunohistochemical images of HER2, gp100, p53, ABCB5, GD2, and TRP2 expression in primary and metastatic melanoma lesions with hematoxylin and eosin (H&E) staining on the left and marker staining on the right.

Representative images for the immunohistochemical expression of the proteins investigated in this study are presented in Figure 1, the association between tumoral expression of the proteins and clinical parameters in Table 1. Among the potential targets in this study, gp100 was the most frequently expressed antigen (61%) in melanoma followed by TRP2 (50%), GD2 (38%), p53 (37%), ABCB5 (17%), and HER2 (3%). Strong IHC expression of TRP2 and gp100 was predominantly confined to the cytoplasm of the tumor cells, whereas p53 expression was limited to the tumor cell nucleus. Positive staining of ABCB5 and GD2 could be found ubiquitously, especially on the melanoma cell membrane and in the cytoplasm. In contrast, HER2 was only found in just a few tumor specimens in advanced melanoma patients with only a faint cytoplasmic HER2 staining.

### 3.3. Correlation of Target Antigen Expression with Clinical Parameters

Comparing the differential expression patterns of the antigens with tumor stages and tumor thickness of the primary, it was found that TRP2 expression was significantly higher in primary melanomas (stage I + II) than in melanoma metastases (stage III + IV) (*p* = 0.005). In addition, TRP2 as well as ABCB5 expression in the primary melanomas was significantly higher in thin tumors (median tumor thickness < 1.5 mm) (TRP2: *p* = 0.013; ABCB5: *p* = 0.025). p53 expression was in tendency higher in ulcerated melanomas (*p* = 0.092). We did not find any significant association between the expression of the target antigens and clinical parameters including gender, age, BRAF mutation status, and serum LDH and S100 levels at the time of biopsy. Besides, the differential expression of these proteins in advanced melanoma patients (stage III and IV) did not reveal any significant effect on patients’ overall survival.

### 3.4. HER2 and GD2 Expression in Primary Melanoma Cell Lines

**Figure 2:**
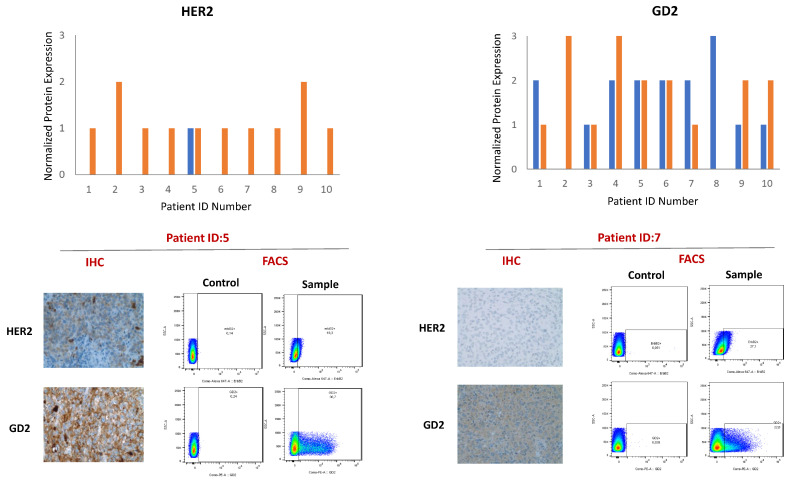
Expression levels of HER2 (left side) and GD2 (right side) in ten cell lines of patients with advanced melanoma. The normalized expression levels (0–3) are indicated in blue for immunohistochemistry (IHC) and in orange for FACS. Representative examples of HER2 and GD2 expression by IHC or FACS are depicted in the lower part.

To confirm the immunohistochemically found surface expression of GD2 and to investigate the rare and confined expression of HER2 in melanoma metastases, we then investigated their expression in ten patient-derived melanoma cell lines grown from fresh melanoma biopsies from the same lesion using flow cytometry. In our study, we observed varying extracellular surface expression of HER2 and GD2 on all patient primary melanoma cell lines. Comparing corresponding IHC data with surface expression detected by FACS analysis revealed for HER2 that even though IHC was positive in only one of the 10 samples, all primary melanoma cell lines derived from the respective tumor sample expressed HER2 in between 5 and 40% of cells. For GD2, expression in the tumor tissue by IHC and in the corresponding melanoma cell line by FACS was more homogenous; however, differing in the amount of expressed antigen (Figure 2).

## 4. Discussion

Tumor antigen-specific redirection of T or NK cells represents a promising therapy against melanoma. Identifying melanoma cell-specific targets remains challenging and is actively being investigated. In line with previous reports, we observed that gp100 was the most frequently overexpressed intracellular protein (61%) and its expression was confined to tumor cells and few melanocytes [31]. Previously, it was shown that both Pmel17 and its highly homologous splice variant gp100 were recognized by melanoma-specific cytotoxic T cells [32]. Furthermore, tebentafusp, an anti-CD3 bispecific fusion protein targeting gp100, was generally well-tolerated and active with a one-year overall survival rate of 65% in both, patients with metastatic uveal melanoma and patients with metastatic cutaneous melanoma in a phase I/II clinical study [5]. Especially in metastatic uveal melanoma, with a poor prognosis of one-year overall survival rate from 13 to 40%, it provides a promising treatment option [33].

Although TRP2 was found to be the second frequently expressed intracellular protein in our observation, its overexpression was more confined to primary melanomas compared to advanced melanoma metastases. Furthermore, in the B16 melanoma model, it was demonstrated that TRP2 TCR transgenic T cells, upon adoptive transfer, can control only pulmonary but not subcutaneous tumors [34]. Similarly, although we did not have an antibody that specifically binds to the mutant p53 protein, p53 overexpression detected with IHC, in general, may reflect the amount of mutant p53. Further, elevated p53 in tumors expressing mutant p53 may also result in the higher presentation of p53-derived peptides by MHC molecules, making it an ideal target for TCR T cell therapy [35,36,37]. However, the large numbers of tumor-associated mutations among the various exons of the p53 tumor suppressor gene reported, make generating a large number of possible mutant p53 vaccines somewhat prohibitive [38].

Among the surface antigens in this study, GD2 was the most promising target for CAR-based T and NK cell strategies. We observed GD2 overexpression in 37% of all our specimens with no difference between the melanoma stages. Furthermore, flow cytometry analysis of patient-derived melanoma cell lines confirmed the abundant extracellular localization of GD2 on the tumor cell surface, suggesting that it can serve as a potential target for active-specific immunotherapy. GD2 specific CAR-T lymphocytes exhibited potent anti-melanoma activity in vitro and in vivo [27]. Meanwhile, a recent study with GD2 directed CAR-T cells along with CD137 as a costimulatory motif demonstrated efficient cytotoxicity against melanoma cells and release of Th1 cytokines in a GD2-specific manner. Interestingly, the study reported in line with our data a positive GD2 staining in 49% of their analyzed melanoma samples but a significant association with tumor stage and overall survival, which could not be confirmed in our data [39]. In a phase I clinical study, four of nine melanoma patients responded to a monoclonal antibody targeting GD2 indicating antitumor activity [40]. CAR-T cells directed to the GD2 antigen in neuroblastoma were proved to induce tumor responses and low-level persistence in patients, which was associated with longer survival [41]. Hence, anti-GD2 CAR-T cells represent a clinically appealing treatment strategy for melanoma patients exhibiting GD2 expression and provide a basis for future studies of the clinical application of immunotherapy for melanoma.

However, GD2 is also expressed in the central nervous system (neuronal cell bodies and mesenchymal stem cells, as well as peripheral nerves) with possible limiting risks and toxicities. Toxicities observed with GD2-targeted antibody therapies included pain, hypertension, urticaria, and complement depletion. Here, a modular universal CAR (UniCAR) platform, which consists of UniCAR-expressing NK cells that cannot recognize target antigens directly but are redirected by a tumor-specific target module (TM) might be a solution [42].

In line with previous reports, we found that HER2 protein expression was very rare, and only 3% of the melanoma samples, all metastases, exhibited a faint IHC staining [43,44]. While this does not rule out possible responses to HER2-targeted immunotherapies as seen with a HER2-specific antibody-toxin in a melanoma patient and HER2-CAR T cells in patient-derived xenograft (PDX) models [29,45], a clear demonstration of HER2 surface expression in primary tumor material appears critical for patient selection. Interestingly, in contrast to our IHC results, the respective cell lines derived from the melanoma tissue samples displayed cell surface extracellular HER2 expression in up to 35% of stained cells in flow cytometry analysis. This might be partly based on methodological differences and differences in the sensitivity of the HER2 antibodies employed for IHC and flow cytometry. Nevertheless, enhanced HER2 levels in the cell lines may also reflect a selective advantage for already HER2-positive cells present at low frequency in the primary tumor tissue or a response of the melanoma cells to the in vitro culture conditions as previously observed for cell lines established from squamous cell carcinomas of the head and neck [46]. Indeed, in contrast to HER2 protein detected by IHC, HER2 mRNA expression appears to be quite common in melanoma [29], suggesting that differential HER2 protein levels in tumor tissues and cell lines may be mainly regulated by post-transcriptional mechanisms. Hence, to judge the potential usefulness of cell-based therapies that specifically target HER2 for subsets of melanomas, more extensive analysis in PDX models may be needed that are directly derived from primary tumor tissues.

In summary, this study shows that within possible targets for cell-based therapies in melanoma including HER2, TRP2, ABCB5, gp100, p53, and GD2, gp100 is the most promising target for TCR-based therapies and GD2 for antibody/CAR-based modalities in melanoma. Limitations of the study are given mainly because of its retrospective nature, the known tissue heterogeneity and possible impact of previous melanoma treatments especially in advanced melanomas. Nevertheless, the study provides an insight into the expression of potential targets for T cell therapy in melanoma and supports the development of gp100 and GD2 targets as monotherapy or in combinations for engineered cell-based therapies against melanoma.

## Figures and Tables

**Table 1:** Expression levels of the antigens TRP2, ABCB5, P53, gp100, and GD2 in correlation with clinical parameters as age, gender, tumor stage, tumor thickness, ulceration of the primary tumor, BRAF V600E/K status, serological LDH and S100 levels.

	All (*n* = 168)	TRP2 (*n* = 141)		ABCB5 (*n* = 137)		P53 (*n* = 139)		gp100 (*n* = 138)		GD2 (*n* = 139)	
	(*n*)	Low (*n*)	High (*n*)	Low (*n*)	High (*n*)	Low (*n*)	High (*n*)	Low (*n*)	High (*n*)	Low (*n*)	High (*n*)
**Age**											
<67	44% (74)	21% (30)	23% (33)	37% (50)	9% (12)	29% (40)	16% (22)	20% (17)	25% (34)	26% (36)	19% (27)
>67	56% (94)	28% (40)	27% (38)	47% (64)	8% (11)	34% (47)	22% (30)	20% (17)	36% (50)	37% (51)	18% (25)
**Gender**											
Male	58% (98)	28% (40)	29% (41)	49% (67)	9% (12)	33% (46)	24% (33)	23% (31)	35% (48)	36% (50)	21% (29)
Female	42% (70)	21% (30)	21% (30)	34% (47)	8% (11)	30% (41)	14% (19)	17% (23)	26% (36)	27% (37)	17% (23)
**Tumor Stage**											
I + II	58% (98)	21% (30)	33% (47)	43% (59)	11% (15)	35% (49)	19% (26)	20% (27)	35% (48)	34% (47)	21% (29)
III + IV	42% (70)	28% (40)	17% (24)	40% (55)	6% (8)	27% (38)	19% (26)	20% (27)	26% (36)	29% (40)	17% (23)
**Tumor Thickness**											
<1.5 mm	46% (77)	16% (23)	29% (41)	33% (45)	12% (16)	31% (43)	14% (19)	18% (25)	28% (38)	27% (38)	19% (26)
>1.5 mm	47% (79)	27% (38)	20% (28)	42% (58)	5% (7)	27% (38)	20% (28)	16% (22)	30% (42)	30% (41)	17% (23)
missing	7% (12)	6% (9)	1% (2)	8% (11)	0% (0)	4% (6)	4% (5)	5% (7)	3% (4)	6% (8)	2% (3)
**Ulceration**											
yes	29% (49)	16% (22)	14% (19)	25% (34)	4% (6)	16% (22)	14% (19)	8% (11)	21% (29)	16% (22)	22% (30)
no	62% (104)	26% (37)	35% (49)	48% (66)	12% (17)	42% (58)	19% (26)	25% (34)	36% (50)	40% (55)	13% (18)
missing	9% (15)	8% (11)	2% (3)	10% (14)	0% (0)	5% (7)	5% (7)	7% (9)	4% (5)	7% (10)	3% (4)
**BRAF V600 E/K**											
yes	17% (28)	12% (17)	6% (9)	15% (21)	3% (4)	12% (16)	7% (10)	9% (13)	9% (13)	12% (17)	9% (13)
no	25% (43)	14% (20)	13% (18)	25% (34)	3% (4)	15% (21)	12% (17)	11% (15)	16% (22)	17% (23)	7% (9)
missing	58% (97)	23% (33)	31% (44)	43% (59)	11% (15)	36% (50)	18% (25)	19% (26)	36% (49)	34% (47)	22% (30)
**LDH**											
normal	70% (117)	38% (53)	32% (45)	58% (79)	13% (18)	42% (59)	27% (37)	28% (38)	42% (58)	45% (62)	25% (34)
elevated	14% (24)	9% (12)	6% (9)	12% (17)	2% (3)	9% (13)	6% (8)	6% (8)	9% (12)	9% (13)	6% (8)
missing	16% (27)	4% (5)	12% (17)	12% (17)	2% (3)	11% (15)	5% (7)	6% (8)	10% (14)	9% (12)	7% (10)
**S100**											
normal	74% (125)	33% (47)	41% (58)	61% (84)	13% (18)	48% (66)	28% (39)	29% (40)	46% (64)	45% (63)	29% (40)
elevated	20% (34)	13% (18)	9% (12)	20% (27)	2% (2)	12% (17)	9% (12)	8% (11)	12% (17)	15% (21)	7% (9)
missing	6% (9)	4% (5)	1% (1)	2% (3)	2% (3)	3% (4)	1 (1%)	2% (3)	2% (3)	2% (3)	2% (3)

## Data Availability

Not applicable.
